# Effects of Allicin on Hypertension and Cardiac Function in Chronic Kidney Disease

**DOI:** 10.1155/2016/3850402

**Published:** 2016-11-21

**Authors:** Ehécatl M. A. García-Trejo, Abraham S. Arellano-Buendía, Raúl Argüello-García, María L. Loredo-Mendoza, Fernando E. García-Arroyo, Mónica G. Arellano-Mendoza, María C. Castillo-Hernández, Gustavo Guevara-Balcázar, Edilia Tapia, Laura G. Sánchez-Lozada, Horacio Osorio-Alonso

**Affiliations:** ^1^Renal Physiopathology Laboratory, Department of Nephrology, Instituto Nacional de Cardiología “Ignacio Chávez”, 14080 Mexico City, Mexico; ^2^Chronic Degenerative Diseases Laboratory, Escuela Superior de Medicina, Instituto Politécnico Nacional, 11340 Mexico City, Mexico; ^3^Departamento de Genética y Biología Molecular, Centro de Investigación y de Estudios Avanzados, Instituto Politécnico Nacional, 07360 Mexico City, Mexico; ^4^Histopathology Laboratory, Research Subdivision, School of Medicine, Universidad Panamericana, Donatello 43, 03910 Mexico City, Mexico; ^5^Multidisciplinary Laboratory, Escuela Superior de Medicina, Instituto Politécnico Nacional, 11340 Mexico City, Mexico

## Abstract

This work was performed to study the effect of allicin on hypertension and cardiac function in a rat model of CKD. The groups were control, CKD (5/6 nephrectomy), and CKD-allicin treated (CKDA) (40 mg/kg day/p.o.). Blood pressure was monitored (weekly/6 weeks). The cardiac function, vascular response to angiotensin II, oxidative stress, and heart morphometric parameters were determined. The CKD group showed hypertension and proteinuria. The coronary perfusion and left ventricular pressures were decreased in CKD group. In contrast, the vascular response to angiotensin II and expression of angiotensin II type 1 receptor (AT1R) were increased. These data were associated with the increment in morphometric parameters (weight of heart and left ventricle, heart/BW and left ventricular mass index, and wall thickness). Concurrently, the oxidative stress was increased and correlated inversely with the expression of Nrf2, Keap1, and antioxidant enzymes Nrf2-regulated. Allicin treatment attenuated hypertension and improved the renal and the cardiac dysfunctions; furthermore, it decreased the vascular reactivity to angiotensin II, AT1R overexpression, and preserved morphometric parameters. Allicin also downregulated Keap1 and increased Nrf2 expression, upregulated the antioxidant enzymes, and reduced oxidative stress. In conclusion, allicin showed an antihypertensive, nephroprotective, cardioprotective, and antioxidant effects, likely through downregulation of AT1R and Keap1 expression.

## 1. Introduction

Chronic kidney disease (CKD) is considered as one of the most important diseases with a great burden on health care systems. As CKD progresses, it often leads to serious cardiovascular (CV) events. The patients with CKD are more likely to die of CV disease (CVD) than to progress to kidney failure [1, 2]. In fact, the risk of CV mortality is 10-fold to 20-fold higher in these patients than in age- and gender-matched control subjects without CKD [3, 4]. There is a strong relationship between CKD and CVD which could be explained by a typical clustering of several risk factors in CKD, such as hypertension, oxidative stress, proteinuria, volume overload, activation of the renin-angiotensin system, and other autocrine and paracrine mechanisms [5]. These factors might be activated independently of hemodynamic overload. With time, abnormalities in both structure and function are observed and occur even at the earliest stages of CKD without a manifest CVD [6]. A fundamental response of cardiomyocyte and left ventricular wall to intrinsic and biochemical stress in CKD is hypertrophy, which at first stages may be successfully compensatory but inevitably will progress to dilation and heart failure.

Despite the variety of drugs for the management of CKD, the cardiovascular complications continue as the leading cause of death in CKD patients. Many studies have documented the beneficial effect of functional foods and nutrients on cardiovascular diseases [7].

Although several reports have suggested that fresh garlic extract has protective actions against cardiovascular disorders, including stroke, coronary artery disease, and hypertension, the precise mechanism responsible for the effects remain to be defined [8–11]. Blood pressure lowering properties of garlic appear to come from the bulb portion of the plant and have been attributed to sulphur-containing compounds. Among these, allicin (allyl 2-propenethiosulfinate or diallyl thiosulfinate) is thought to be one of the main bioactive, organosulfur compounds synthesized in garlic. Allicin is naturally produced from the stable precursor S-allyl cysteine-S-oxide (alliin) by the action of the enzyme alliinase when garlic cloves are crushed or macerated [12].

Recent studies have shown that allicin treatment reduces systemic blood pressure in spontaneously hypertensive rats and protects against coronary endothelial dysfunction and right heart hypertrophy in hypertensive rats [12, 13]. Despite the studies supporting the vasoprotective effects of garlic, the role of allicin on cardiac function in CKD still remains unknown. Therefore, the present study was aimed at investigating the effects of allicin on hypertension and cardiac function in an experimental model of CKD.

## 2. Material and Methods

### 2.1. Reagents

Angiotensin II (Ang II), dichloromethane, diallyl disulphide, hydrogen peroxide, 4-nitrophenol, tetramethoxypropane, dinitrophenylhydrazine, methanesulfonic acid, and 1-methyl-2-phenylindole were purchased from Sigma-Aldrich (St. Louis, MO, USA). All other chemicals used were of the highest purity available.

### 2.2. Experimental Design

Male Wistar rats weighing 280–300 g of body weight were used. The rats were randomly distributed into three groups: control or sham (C), chronic kidney disease (CKD) [induced by subtotal renal ablation (5/6Nx)], and chronic kidney disease allicin-treated (CKDA) (40 mg/kg/day, p.o.). All experimental groups were maintained under laboratory diet, water* ad libitum*, 12-hour light/dark cycles, and temperature at 21°C and relative humidity at 70% for six weeks.

### 2.3. Ethics Statement

This study was performed in accordance with the Guide for the Care and Use of Laboratory Animals, published by the US National Institutes of Health, and approved by the Research Committee of the National Institute of Cardiology I. Ch. and by the Mexican Federal Regulation for animal experimentation and care (NOM-062-ZOO-2001) and for the disposal of biological residues (NOM-087-ECOL-1995).

### 2.4. Allicin

Allicin was produced by oxidation of diallyl disulphide as previously reported [14]. In brief, 1 g diallyl disulphide was dissolved in 5 mL acetic acid under stirring in an ice bath. Hydrogen peroxide (1.5 mL, 30% v/v) was added stepwise and the reaction was allowed to proceed for 30 min. Afterwards, the reaction was kept at 13°C for 20 min; then, it was put again in ice bath for 5 h, stopped with 15 mL distilled water at pH 6.5, and extracted with 30 mL dichloromethane. After 5 extractions with 5% (w/v) Na_2_CO_3_ (20 mL each) and 3 extractions with distilled water (20 mL each), the solvent was left to evaporate until yellowish oil (allicin) remained. For stabilization and storage, allicin was resuspended in water at 2.5% (w/v) and kept at −70°C until used.

### 2.5. Induction of Experimental Model of Chronic Kidney Disease

Under anesthesia with isofluorane, subtotal renal ablation was performed by removal of the right kidney and selective infarction of approximately two-thirds of the left kidney by ligation of two or three branches of the renal artery (5/6Nx). Sham operation consisted of ventral laparotomy and manipulation of the kidneys and renal pedicle without destruction of renal tissue. The muscle and skin incisions were sutured with polypropylene suture. The animals were returned to the animal facility for surgery recovery [15].

### 2.6. Systolic Blood Pressure Record (SBP)

After 5/6Nx, the systolic blood pressure (SBP) was monitored weekly during six weeks by a validated tail-cuff plethysmography method using conscious rats (XBP-1000 Kent Scientific, CT, USA).

### 2.7. Renal Function

At six weeks of follow-up, rats were placed in metabolic cages (Nalgene, Rochester, NY, USA) to collect 24 h urine. Urine samples were centrifuged at 5000 ×g for 15 minutes to remove debris, and the supernatant was analyzed. The urinary variables measured were diuresis, creatinine, urea (IL 300 plus, Clinical Chemistry Analyzer, Instrumentation Laboratory, Bedford, MA, USA), and proteinuria.

### 2.8. Assessment of Cardiac Function

The rats were anesthetized intraperitoneally with sodium pentobarbital (60 mg/kg) and the complete lack of pain response was assessed by determining pedal withdrawal reflex. Then, sodium heparin was injected (1000 U/kg) and 5 min later a midsternal thoracotomy was performed. The chest was opened, and a loose ligature was passed through the ascending aorta. The heart was trimmed of noncardiac tissue and perfused in a retrograde fashion via a nonrecirculating perfusion system at constant flow. Coronary flow was adjusted with a variable-speed peristaltic pump (Harvard Apparatus, model 1215 Holliston, MA, USA). An initial perfusion rate of 15 mL/min for 5 min was followed by a 25 min equilibration period at a perfusion rate of 8 mL/min. The perfusion medium was Krebs-Henseleit solution with the following composition (mM): NaCl 117.8; KCl 6; CaCl_2_ 1.75; MgSO_4_ 1.2; NaH_2_PO_4_ 1.2; NaHCO_3_ 24.2; glucose 5; and sodium pyruvate 5. The solution was equilibrated with 95% O_2_–5% CO_2_, pH 7.4, and kept at 37.5°C. A pair of stimulating electrodes, made of small stainless steel wire (vascular clamps/Fine Surgical Instruments, CA, USA), were placed 2 mm apart in the right atrioventricular groove. Pacing was achieved by applying electrical square pulses of 2 ms and at twice the electrical threshold at a rate of 4.5 ± 0.3 Hz.

After equilibration period, to determine the variations in coronary perfusion and systolic pressure, increments in coronary flow were performed (6–14 mL/min).

To measure left intraventricular pressure, a latex fluid filled balloon (400 *μ*L Krebs solution) was placed in the ventricle and connected to a pressure transducer, adapted in turn, to a computerized data acquisition system (BIOPAC Systems, CA, USA). Measurements of LV function were obtained when the preparation achieved a steady state (≈15 min).

To assess coronary reactivity in heart, a dose-response assay was evaluated at increasing doses of Ang II (10^−9^, 10^−8^, 10^−7^, and 10^−6^ M) for 10 minutes via a flow pump connected to the perfusion cannula.

### 2.9. Heart Morphology and Histology

At the end of the follow-up, six rats of each experimental group were anaesthetized with intraperitoneal injection of pentobarbital sodium (40 mg/kg). A polyethylene catheter filled with phosphate buffer (0.2 M, pH 7.4) and heparin (100 IU/mL) was introduced into the ascending aorta. Then, the heart was arrested in diastole by injecting 1.0 mL of cadmium chloride (100 mM) through the aortic catheter and was perfused retrogradely with phosphate buffer for 3 min, followed by perfusion with 10% neutral buffered formalin solution for 15 min. After perfusion-fixation, the heart was excised and atria and right ventricle were dissected from the LV and all pieces weights were recorded. The LV was cut into three transverse slices; the slice halfway between the base and the apex was embedded in paraffin, sectioned at 4 *μ*m intervals, and stained with HE [16].

Of each heart, four digital photomicrographs were obtained with the low power objective (5x) and, by an image analysis program (Axiovision Rel 4.8.2, Zeiss, Germany), the free wall thickness was calculated. The LVMI was defined as the value of the left ventricular weight (mg) divided by the total body weight (g).

### 2.10. Evaluation of Oxidative Stress in Heart

*Lipid Oxidation*. 4-Hydroxynonenal (4-HNE) was measured using a standard curve of tetramethoxypropane. A solution of 1-methyl-2-phenylindole in acetonitrile : methanol (3 : 1) was added to heart homogenates and the reaction was started with 37% HCl or methanesulfonic acid plus FeCl_3_, to measure 4-HNE, respectively. Optical density was measured at 586 nm after 1 h of incubation at 45°C. Data were expressed as nanomoles of 4-HNE per milligram of protein.

*Oxidized Proteins*. The determinations of carbonyl groups in the proteins were measured using the reaction with 2, 4-dinitrophenylhydrazine (DNPH). Carbonyl groups were estimated by using the molar absorption coefficient of 22,000 M^−1^·cm^−1^ for DNPH derivatives, and its concentration was expressed as nmol carbonyl groups/mg protein.

### 2.11. Expression of Angiotensin II Type 1 Receptor (AT1R), Nuclear Factor (Erythroid-Derived 2) Like 2 (Nrf2), and Kelch-Like ECH-Associated Protein 1 (Keap1) in Heart

The frozen heart was ground to a powder and then mixed in ice-cold HEPES buffer (10 mM HEPES, 0.2% Triton X-100, 50 mM NaCl, 0.5 mM sucrose, 0.1 mM EDTA, protease, and phosphatase inhibitors) and homogenized with an ice-chilled Dounce homogenizer at 4°C. An aliquot of the homogenate was stored and the rest was used to make cytosolic and nuclear extracts. This was spun at 10,000 rpm for 10 min, and the supernatant was aliquoted and stored at −70°C as the cytosolic extract. The pellet was suspended in ice-cold buffer (10 mM HEPES, 500 mM NaCl, 10% glycerol, 0.1 mM EDTA, 0.1 mM EGTA, 0.1% IGEPAL, and protease and phosphatase inhibitors) and vortexed at 4°C for 15 min and centrifuged for 10 min at 14,000 rpm. The resulting supernatant was aliquoted and stored as the nuclear extract at −70°C. A small aliquot of heart homogenate, cytosolic, and nuclear extract was kept at 4°C for protein estimation by the Bradford method using bovine serum albumin as standard. Thirty *μ*g of total protein was used to evaluate expression of AT1 receptor, Nrf2, and Keap1. Nuclear extracts were used for Nrf2 immunoblotting. Intensity values were normalized to a loading control (housekeeping) and expressed as arbitrary units of protein expression. Glyceraldehyde-3-phosphate dehydrogenase (GAPDH) and proliferating cell nuclear antigen (PCNA) were used as a loading control (housekeeping).

### 2.12. Immunofluorescence

Immunofluorescence analysis of Nrf2 was performed using 4 *μ*m sections from at least 2 animals per group, paraffin embedded renal tissue. After deparaffinization and rehydration, the samples were submitted to heat induced antigen retrieval in sodium citrate buffer (10 mM, pH 6.0) during 20 minutes. Next steps were as follows: incubation during 1 hour with a blocking solution (2% bovine serum albumin, 5% normal goat serum), 1 hour with the primary antibody (anti-Nrf2 rabbit polyclonal GeneTex GTX 103322) at a 1/50 dilution, and 1 hour with a secondary FITC-conjugated goat anti-rabbit antibody at a 1/100 dilution; all these reagents were diluted in TBS with 0.05% Tween® 20. Then, the Tyramide Signal Amplification fluorescence kit from Perkin Elmer (NEL741E001KT) was used according to manufacturer instructions. Briefly, an anti-fluorescein HRP conjugated antibody was applied to the sections (30 min), followed by incubation for 3 minutes with the amplification reagent. All the steps were performed at room temperature and the slides were washed with TBS with 0.05% Tween 20 after each step, except after the application of the blocking solution. For nuclear counterstaining, DAPI was used. The same microscope and software mentioned above for the histological assessment were utilized for the fluorescence labeled sections analysis. Microscopic images were obtained using a digital camera mounted on a light microscope (Axiophot 2, Zeiss, Germany).

### 2.13. Statistical Analysis

All data are presented as means ± SEM. Comparisons among group were made using one-way analysis of variance followed by Tukey's test by using GraphPad version 5.00 (GraphPad Software, La Jolla, CA, USA). Results were considered significant when *p* < 0.05.

## 3. Results

At the beginning of the experiments, differences in the body weights among rats in all experimental groups were indistinguishable. However, at six weeks of follow-up, the body weight average was significantly lower in CKD group as compared with control group (Table 1). The allicin treatment favored weight gain when compared to the CKD untreated group

### 3.1. Renal Function

To validate the CKD model, renal function was evaluated through measurement of creatinine and blood urea nitrogen (BUN) in serum and urine, as well as diuresis and proteinuria. The CKD group showed statistically significant higher levels of creatinine and BUN in serum and urine and increased diuresis and proteinuria in comparison to those of the control group (Table 1).

Allicin treatment improved renal function evidenced by a significant reduction in creatinine and BUN levels in serum and urine as well as in proteinuria when compared with CKD group (Table 1).

### 3.2. Blood Pressure

**Figure 1:**
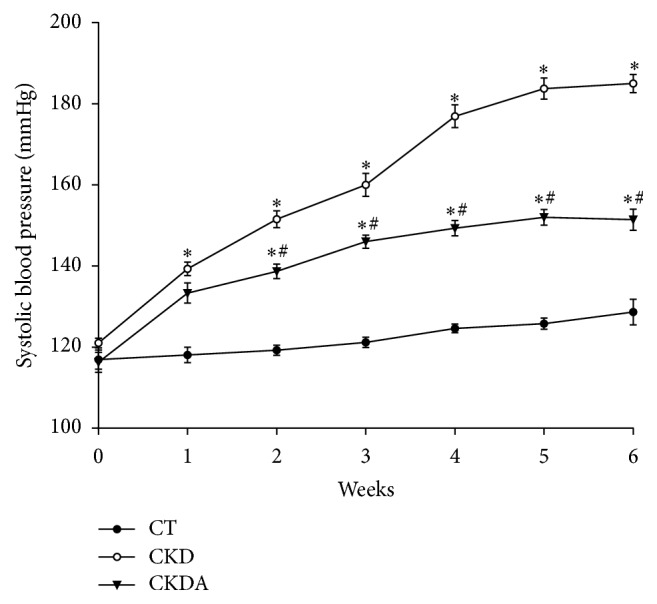
Systolic blood pressure in experimental groups by tail-cuff method. CT: control; CKD: chronic kidney disease; CKDA: chronic kidney disease treated with allicin. Values are expressed as mean ± SD of at least 6 animals from each experimental group, ^*∗*^ *p* < 0.05 versus CT and ^#^ *p* < 0.05 versus CKD.

Renal chronic disease is frequently associated with high blood pressure; therefore, we measured systolic blood pressure in the experimental groups by tail-cuff method in conscious rats. At baseline, rats from all experimental groups had similar systolic blood pressure (Figure 1). However, at six week of follow-up, the group with CKD showed significantly higher systolic blood pressure as compared with control group. The systolic blood pressure was increased in a time-dependent fashion and at the end of the study reached ≈180 mmHg ± 6 mmHg. In contrast, control group remained with a blood pressure within normal range (Figure 1). Interestingly, allicin treatment partially but significantly prevented the increase in systolic blood pressure from the second week to the end of the study; thus rats in this group reached 140 mmHg at week 6, 40 mmHg less compared to CDK group without treatment (Figure 1).

### 3.3. Assessment of Cardiac Function In Vitro

To evaluate cardiac function in the various experimental groups, we determined the coronary perfusion pressure and left ventricular systolic pressure.

**Figure 2:**
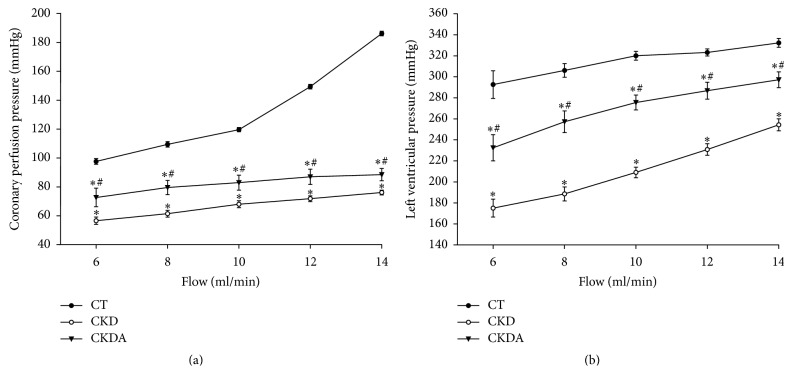
(a) Flow-dependent coronary perfusion pressure and (b) Flow-dependent left ventricular pressure. CT: control; CKD: chronic kidney disease; CKDA: chronic kidney disease treated with allicin. Values represent mean ± SD of at least 6 animals from each experimental group. ^*∗*^ *p* < 0.05 versus CT and ^#^ *p* < 0.05 versus CKD.

The coronary perfusion pressure flow relationship for heart is summarized in Figure 2(a). Control group depicted the normal heart response; thus increments in coronary flow rate from 6 to 10 mL/min resulted in a proportional raise in coronary perfusion pressure; then an abrupt increment in perfusion pressure was obtained at flows of 12 and 14 mL/min in this group. In contrast, CKD group showed a significantly blunted response, demonstrated by a flattened coronary perfusion pressure flow-dependent curve compared to control group (Figure 2(a)). Allicin treatment improved coronary perfusion pressure as compared to the CKD untreated group, although the effect was clearly appreciated from basal to hypervolemic coronary flow conditions (Figure 2(a)).

The left ventricular pressure response to increase in coronary flow rate in the different groups is shown in Figure 2(b). The left ventricle pressure of CKD group increased in a flow-dependent manner. However, the left ventricular performance of these hearts was significantly lower than those of the control group (Figure 2(b)). Regarding the CKD-allicin treated group, it showed an improvement in the left ventricular performance when compared with CKD group untreated, without reaching the normal values. However, the isolated heart from CKDA group showed a better response to variable coronary flow rate, from hypovolemic to hypervolemic conditions (Figure 2(b)).

### 3.4. Effect of Allicin on Vascular Reactivity to Angiotensin II and AT1R Expression

**Figure 3:**
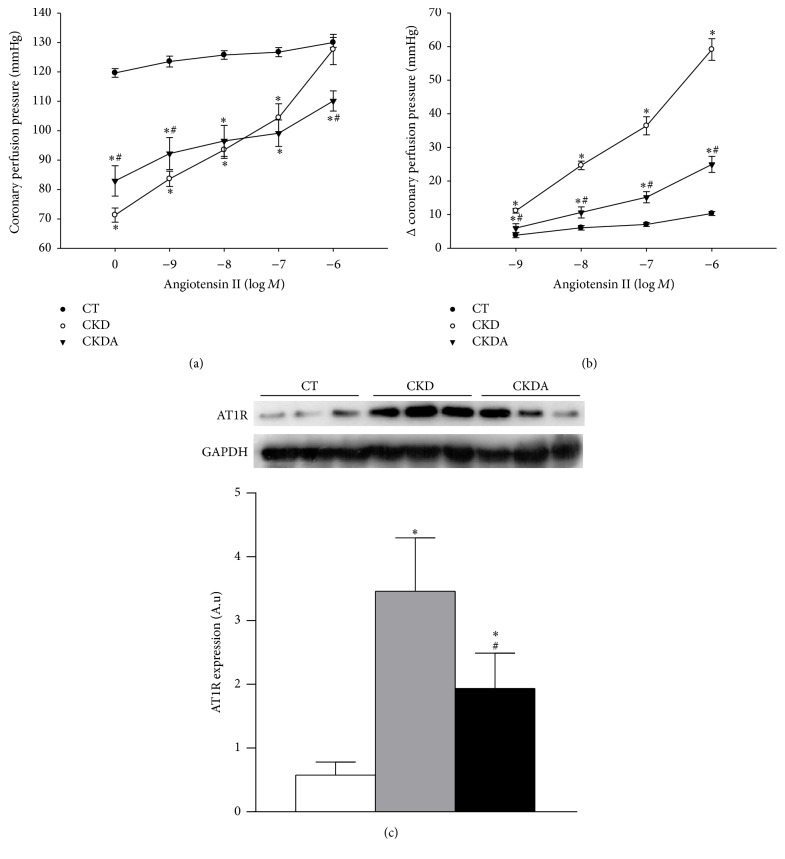
Effect of allicin treatment on (a) vascular reactivity to angiotensin II, (b) changes (Δ) of coronary perfusion pressure, and (c) angiotensin II type 1 receptor (AT1R) expression in heart. CT: control; CKD: chronic kidney disease; CKDA: chronic kidney disease treated with allicin. Values represent mean ± SD of at least 6 animals from each experimental group. ^*∗*^ *p* < 0.05 versus CT and ^#^ *p* < 0.05 versus CKD.

To assess vascular reactivity, perfused isolated hearts were stimulated with Ang II in a concentration-response curve. The Ang II infusion induced increases in coronary perfusion pressure in a concentration-dependent fashion in all experimental groups (Figure 3(a)). However, in the isolated heart from CKD group, the stimuli with Ang II increased coronary perfusion pressure significantly between each point [log⁡*M*] compared with response observed in control group (Figure 3(a)), even at very low concentrations (from 0 to −9 [log⁡*M*]). In the CKDA group, the Ang II concentration-response assay showed an increase in coronary perfusion pressure in a concentration-dependent way, but these increments were lower in CKDA than those observed in the CKD untreated group (Figure 3(a)). The effects induced by Ang II stimuli as well as the cardioprotective effect of allicin on the cardiac response to Ang II are clearly appreciated in Figure 3(b).

To better understand the mechanism involved in the improvement of the cardiovascular response to Ang II, we evaluated the protein level expression of Ang II type 1 receptor (AT1R) in heart. In the CKD group, the AT1R expression was increased compared with that of the control group. Allicin treatment partially decreased AT1R expression in comparison to that of the CKD untreated group (Figure 3(c)).

### 3.5. Measurement of Morphometric Variables

**Figure 4:**
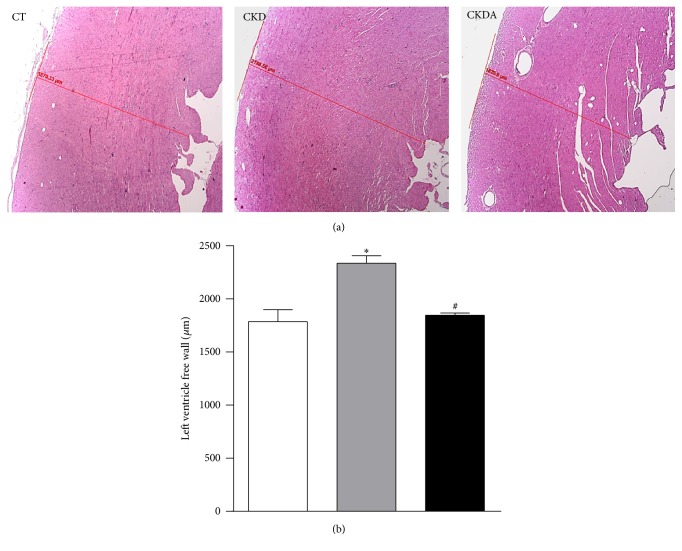
Left ventricle free wall portion photomicrograph (a) and quantitative analysis (b). CT: control; CKD: chronic kidney disease; CKDA: chronic kidney disease treated with allicin. Values represent mean ± SD. ^*∗*^ *p* < 0.05 versus CT and ^#^ *p* < 0.05 versus CKD.

Upon gross observation, rats in the CKD group had visibly hypertrophic hearts compared with the control group. In this study, we did not observe differences in right atrium, left atrium, and right ventricle weights. However, a significant increase in heart and left ventricle weight, LVMI, and heart/BW index were observed in CKD group when compared with those of the control group (Table 2), suggesting the presence of left ventricular hypertrophy. Interestingly, the increase in left ventricle weight and LVMI in the CKD group was associated with global LV wall thickening (Figure 4), a sign of CVD [5]. The mean thickness LV free wall was significantly higher in CKD group than that of the control group (*p* < 0.05). Importantly, allicin treatment prevented all morphologic alterations observed in heart samples from the CKD group such as the increment in LVMI, left ventricular weight, and wall thickness of heart (Table 2 and Figure 4).

### 3.6. Evaluation of Oxidative Stress in Heart

Oxidative stress is one of multiple molecular mechanisms involved in the etiology of hypertension, CVD, and CKD.

**Figure 5:**
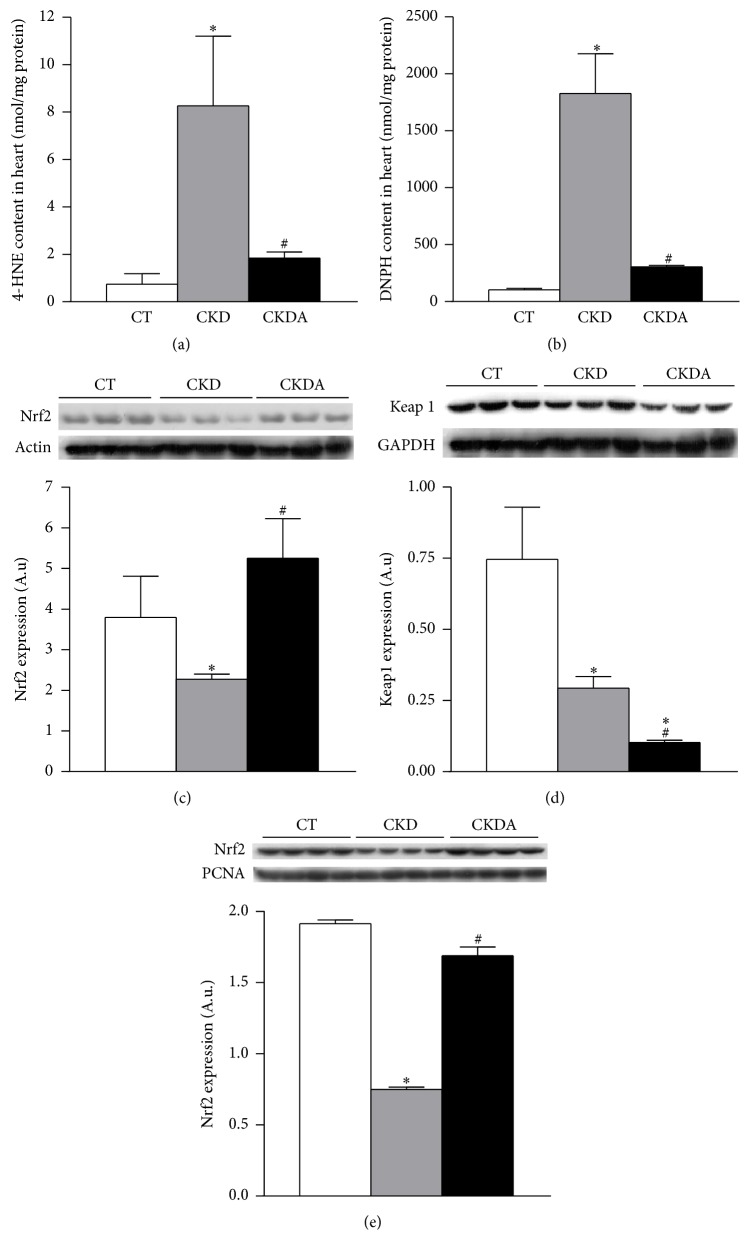
Evaluation of oxidative stress in heart. (a) Lipid oxidation, (b) protein oxidation, (c) Nrf2 expression, (d) Keap1 expression, and (e) nuclear expression of Nrf2. CT: control; CKD: chronic kidney disease; CKDA: chronic kidney disease treated with allicin. Values are resented as mean ± SD of at least 6 animals from each experimental group. ^*∗*^ *p* < 0.05 versus CT and ^#^ *p* < 0.05 versus CKD.

To assess oxidative stress in heart during CKD, lipid and protein oxidation were evaluated by measuring tissue content of 4-HNE and carbonylated proteins, respectively. In whole heart, lipid and protein oxidation were significantly increased in CKD group when compared to the control group (Figures 5(a) and 5(b)). This CKD-induced cardiac oxidative stress was decreased by allicin treatment (Figures 5(a) and 5(b)).

The expressions of Nrf2 and Keap1 were also assessed in total heart tissue, as this pathway is the master regulator of the antioxidant cellular protection. After six weeks of follow-up, in heart from group with CKD, the Nrf2 and Keap1 protein expression was lower than that of the control group (Figures 5(c) and 5(d)). The allicin treatment restored the Nrf2 protein expression levels (Figure 5(c)). Additionally, allicin decreased the Keap1 protein expression compared with group with CKD untreated and control group (Figure 5(d)).

**Figure 6:**
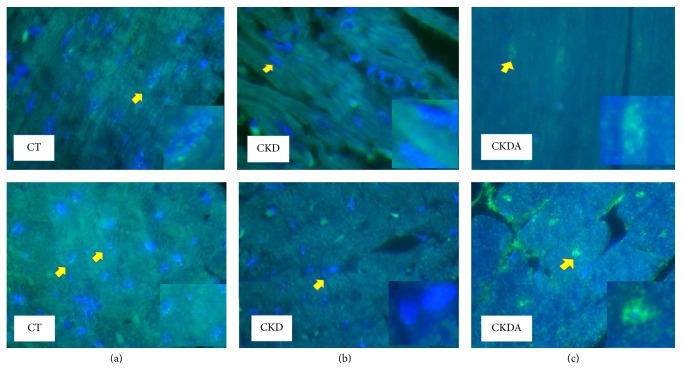
Localization and expression of Nrf2 in heart. (a) Control (CT), (b) chronic kidney disease (CKD), and (c) chronic kidney disease-allicin (CKDA) treated. Representative heart sections micrographs showing immunoreactivity for Nrf2. Upper row longitudinal areas and lower row cross areas. Left column, control group depicting scarce positivity in nuclei; middle column, CKD group, with no evidence of reactivity; and right column, CKD-allicin treated group with intense reactivity in nuclei. Original magnification 100x.

After these observations, to determine if transcription mechanism was involved in the cardioprotective effect of allicin, we assessed the Nfr2 expression using a nuclei-enriched fraction. The expression of Nrf2 in the CKD group was lower compared to the control group. This effect CKD-induced was prevented by treatment with allicin (Figure 5(e)). These results were supported by microscopy analysis using immunofluorescence (Figure 6). The immunofluorescence labeling showed a control group depicting scarce positivity in nuclei. In the CKD group, we did not observe clear evidence of Nrf2 immunoreactivity compared with control group. In contrast, in the CKD-allicin treated group was noticeable intense reactivity in nuclei compared with the CKD untreated (Figure 6).

**Figure 7:**
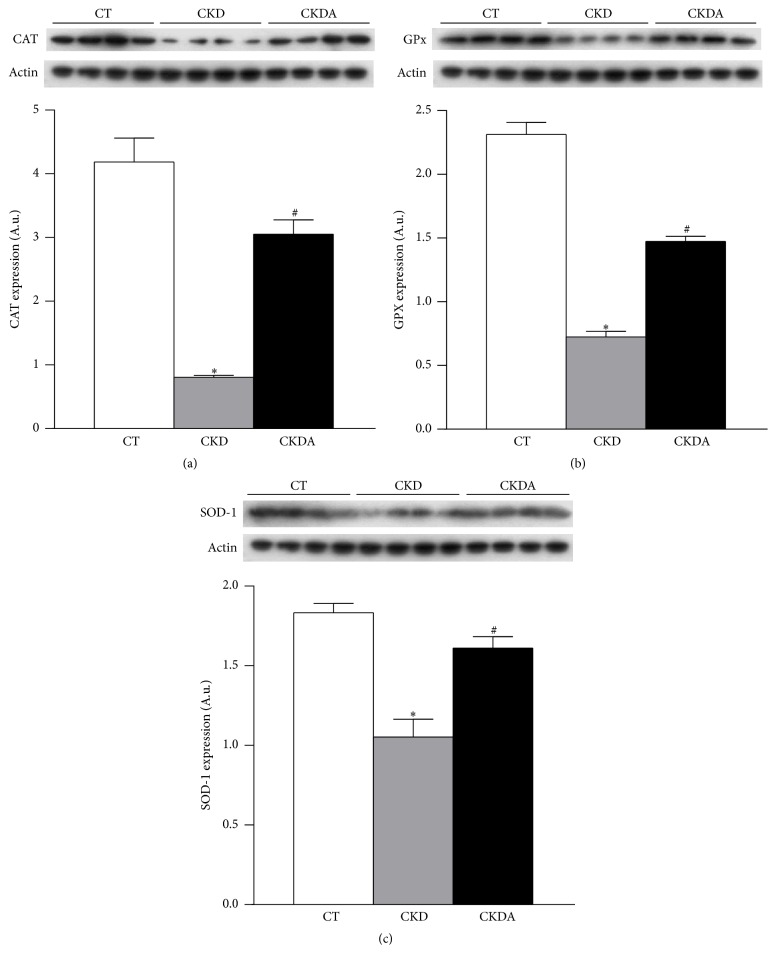
Evaluation of antioxidant enzymes Nrf2-regulated in heart. (a) Catalase (CAT), (b) glutathione peroxidase (GPx), and (c) superoxide dismutase (SOD). CT: control; CKD: chronic kidney disease; CKDA: chronic kidney disease treated with allicin. Values are presented as mean ± SD of at least 5 animals from each experimental group. ^*∗*^ *p* < 0.05 versus CT and ^#^ *p* < 0.05 versus CKD.

Thus, we hypothesize that Nrf2 would stimulate the expression of its target genes. Therefore, we assessed the expression of endogenous antioxidant enzymes CAT, SOD, and GPx. The protein expression of CAT, SOD, and GPx was decreased in cardiac tissue from CKD group compared with control group. The allicin treatment restores this CKD-induced effect (Figure 7).

## 4. Discussion

In the present study, we evaluated the therapeutic effect of allicin on hypertension and cardiac function in a rat model of CKD. We provide evidence that allicin improved renal function, partially decreased systemic hypertension, improved the function of the heart, and prevented its remodeling, in addition to almost abolishing the oxidative stress. The protective effects of allicin were mediated by reducing the AT1R and Keap1 expression. In contrast, allicin increased the Nrf2 expression and consequently their target genes (CAT, SOD, and GPx), which led to decrease in hypertension, oxidative stress, ultimately improving cardiac structure and performance.

The beneficial effects of garlic against cardiovascular disorders such as stroke, coronary artery disease and hypertension are well known [8–11]. It has been suggested that the active substance in garlic may be one of the sulphur compounds; among them, allicin is considered as one of the highest bioactive organosulphur compounds from garlic [12]. Despite the fact that the effect of whole garlic extract on hypertension is well known, the effects of allicin on blood pressure and renal and cardiac function specifically in CKD are scarce [17–19]. Thus, this study was designed to assay the usefulness of allicin in treatment of complications associated with CKD.

In our study, allicin improved the biochemical markers of renal dysfunction such as creatinine, blood urea nitrogen, diuresis, and proteinuria. To our knowledge, this is the first study showing the nephroprotective effects of allicin on an experimental model of CKD. In addition, treatment with allicin in a control group did not affected neither blood pressure nor renal function (data not shown). The CKD group showed a blood pressure progressive increment longitudinally in time, which allicin treatment reduced significantly from the early stages to the end of study. In agreement with our results, chronic oral administration of allicin reduced systolic blood pressure in different conditions such as spontaneously hypertensive rats, hypercholesterolemic rats, and fructose-induced hyperinsulinemic hyperlipidemic rats [12, 20, 21].

Our results on cardiac function showed a decrement in coronary perfusion pressure and left ventricular performance in CKD group. These results indicate a dysfunctional heart unable to adapt to pumping sufficient amounts of blood to meet acute metabolic demands of organs and tissues, for example, during physical exercise. This effect causes exercise intolerance, impairs quality of life, and has been associated with high morbidity and mortality. In contrast, the vascular response to Ang II was increased and this result was associated with the increased AT1 receptor expression in heart from CKD group. These data indicates hyperreactive cardiac endothelium, mainly to vasoconstrictors such as Ang II. In addition, our data from morphometric analysis suggest cardiac hypertrophy. It has been reported that the increased Ang II receptors contribute to the growth of the heart and hence to maintaining hypertrophy [22]. Abnormalities in both cardiovascular structure and function are observed early in the course of CKD [6], with the left ventricular hypertrophy as the most common abnormality in adult patients [5].

The alterations in cardiac function, vascular response to Ang II, AT1 receptor expression and morphometric analysis were attenuated by the allicin treatment. Previous studies demonstrated that garlic oil attenuates the impaired cardiac contractility [23, 24]. Other studies reported that allicin protected cardiac function and prevented the development of cardiac hypertrophy through ROS-dependent mechanism involving multiple intracellular signaling (ERK1/2, JNK1/2 and PI3/Akt/GSK3*β* signaling pathways) [25].

On the other hand, it has been described that allicin has vasodilator activity independent of the synthesis of nitric oxide, ATP-sensitive K(+) channels, activation of cyclooxygenase enzyme, or changes in bronchomotor tone in the pulmonary vascular bed of the rat; meanwhile, its different metabolites do not possess this effect [26, 27]. There is a possibility that allicin may act through another mechanism, specifically through the inhibition of the renin-angiotensin system which plays a significant role in renovascular hypertension. Garlic lowers blood pressure through the reduction in angiotensin converting enzyme activity (ACE) [28], and the effects of vasoconstrictors simultaneously increase the effects of vasodilators. In the 2 K-1C hypertensive rat model, a significant reduction in SBP was noted in garlic-treated rats. This decrease correlates positively with reduction in ACE activity in the serum, aorta, heart, lung, and kidney [29]. However, the capacity of garlic to downregulate the AT1R expression only has been described in streptozotocin-induced diabetic rats [30].

Increased oxidative stress in heart from CKD group was evaluated by measuring lipid and protein oxidation, which was correlated with the lower nuclear translocation and expression of Nrf2 a redox-sensitive transcription factor [31]. The allicin treatment decreased the oxidative stress via an increase in Nrf2 expression and decreasing the expression of its repressor Keap1. Moreover, allicin stimulates Nrf2 nuclear translocation and the expression of antioxidant enzymes CAT, SOD, and GPx.

The exact mechanism through which allicin exerts its antioxidant effects has not yet been fully elucidated. Some researchers have suggested that allicin exerts direct antioxidant effects by preventing the formation of free radicals [32–34], the lipid peroxidation through scavenge OH molecules, and the chain-carrying peroxyl radicals of the substrates by transferring its allylic hydrogen to the oxidized substrate [35, 36]. Allicin also acts as an indirect antioxidant by inducing increased levels of intracellular glutathione level via the upregulation of phase II detoxifying enzymes in a Nrf2-dependent pathway (hemeoxygenase-1, superoxide dismutase, glutathione peroxidase, glutathione-S-transferases, NAD(P)H-quinine oxidoreductase, and *γ*-glutamylcysteine synthetase) [13, 37, 38].

Our data shows that allicin downregulates AT1 and Keap1 possibly as the main mechanism, cardioprotective mechanism. However, we cannot discard that the beneficial effects of allicin may be mediated by an improved endothelial function, vasodilatory effects, downregulation of AT1 receptor, and hence reduction in AT1 activity and by an antioxidant mechanism by itself, or by indirectly stimulating the degradation of Keap1. Therefore, the inhibition of the interaction between Nrf2 and Keap1 favors nuclear Nrf2 translocation to target gene transactivation [39]. All these effects collectively contributed to the beneficial effect of allicin on the heart in CKD.

At our knowledge, this is the first study showing the effects of allicin on cardiac function, on vascular reactivity to Ang II, and on the expression of AT1R and Keap1 in the heart during the experimental CKD. Our findings highlight the potential of allicin to ameliorate the heavy cardiovascular burden in patients with chronic renal failure (CRF). It is known, for example, that hemodialysis contributes to increasing free radical production and to diminishing antioxidant defenses in CRF patients. In this regard, a recent study demonstrated that oxidative stress was the principal risk factor for cardiovascular mortality in a population of elderly patients undergoing hemodialysis and followed up for 4 years [40]. Furthermore, because heart dysfunction secondary to CRF is a common event, allicin administration could alleviate cardiac complications in some patients.

This study has some limitations. The main limitation is the short bioavailability of allicin. Allicin has a short half-life and it is disintegrated to other compounds such as diallyl sulfide, disulfides, trisulfides, and ajoene [41]. Allicin cannot be detected in the blood or urine after ingestion of raw garlic or pure allicin [42]. To date, there is no assay to measure plasma levels of allicin; thus the correlation with the effects of allicin cannot be ascertained. Finally, whether low doses of allicin than those used in our study can be effective in reducing the blood pressure using a less aggressive experimental model even more chronic still needs to be investigated. Thus, more clinical and animal studies to validate allicin potential use for hypertension management are required.

In conclusion, allicin showed nephroprotective, antihypertensive, cardioprotective, and antioxidant effects, likely mediated by the downregulation of angiotensin II type 1 receptor and the Nrf2-inhibitor Keap1. Furthermore, our data provide evidence supporting the use of allicin as therapy in pathophysiological conditions in which cardiac and/or renal functions may be involved or associated.

## Figures and Tables

**Table 1:** Body weight and renal function parameters in the experimental groups studied.

	Sham	CKD	CKDA
Body weight (g)	386 ± 4.59	327.44 ± 2.95^*∗*^	360.33 ± 1.85^*∗*#^
Serum creatinine (mg/dL)	0.41 ± 0.01	1.42 ± 0.10^*∗*^	1.08 ± 0.04^*∗*#^
Blood urea nitrogen (mg/dL)	3.8 ± 0.30	56.11 ± 2.71^*∗*^	43.9 ± 2.43^*∗*#^
Urinary volume (mL/24 h)	19.92 ± 1.1	32.86 ± 3.36^*∗*^	45.58 ± 4.35^*∗*#^
Urinary creatinine (mg/dL)	34.6 ± 2.74	42.92 ± 3.56^*∗*^	35.59 ± 3.42^#^
Urinary urea (mg/dL)	115 ± 8.50	177.6 ± 8.80^*∗*^	180.1 ± 10.84^*∗*^
Proteinuria (mg/24 h)	12.32 ± 0.95	232.15 ± 23.19^*∗*^	125.29 ± 13.1^*∗*#^

CKD: chronic kidney disease; CKDA: chronic kidney disease treated with allicin. Data are expressed as mean ± SEM of at least 6 animals from each experimental group. ^*∗*^ *p* < 0.001 versus control and ^#^ *p* < 0.001 versus CKD.

**Table 2:** Gross cardiac measurements.

	CT	CKD	CKDA
Right atrium (mg)	26.6 ± 2.5	31.67 ± 2.2	29.67 ± 2.9
Left atrium (mg)	24 ± 4.5	24.89 ± 1.48	23.33 ± 2.01
Right ventricle (mg)	263 ± 20.9	272 ± 12.08	239.7 ± 25.94
Left ventricle (mg)	726.8 ± 21.27	1025 ± 41.04^*∗*^	834 ± 38.67^*∗*#^
Heart (g)	1.04 ± 0.02	1.35 ± 0.04^*∗*^	1.12 ± 0.08^*∗*#^
LVIM (mg/g)	1.88 ± 0.03	3.12 ± 0.1^*∗*^	2.3 ± 0.1^*∗*#^
Heart/BW (g)	2.69 ± 0.04	4.13 ± 0.11^*∗*^	3.12 ± 0.17^*∗*#^

CT: control; CKD: chronic kidney disease; CKDA: chronic kidney disease treated with allicin; LVMI: left ventricle mass index; BW: body weight. Data are expressed as mean ± SEM of at least 6 animals from each experimental group. ^*∗*^ *p* < 0.001 versus control and ^#^ *p* < 0.001 versus CKD.
